# *Mycobacterium avium* Subsp. *hominissuis* Interactions with Macrophage Killing Mechanisms

**DOI:** 10.3390/pathogens10111365

**Published:** 2021-10-22

**Authors:** Norah Abukhalid, Sabrina Islam, Robert Ndzeidze, Luiz E. Bermudez

**Affiliations:** 1 Department of Biomedical Sciences, College of Veterinary Medicine, Oregon State University, Corvallis, OR 97331, USA; norah.abukhalid@oregonstate.edu (N.A.); sabrina.islam@oregonstate.edu (S.I.); robert.ndzeidze@oregonstate.edu (R.N.); 2 Department of Microbiology, College of Science, Oregon State University, Corvallis, OR 97331, USA

**Keywords:** *Mycobacterium avium* subspecies *hominissuis*, nitric oxide, host-pathogen interface, amoeba and macrophage, reactive oxidative radicals

## Abstract

Non-tuberculosis mycobacteria (NTM) are ubiquitously found throughout the environment. NTM can cause respiratory infections in individuals with underlying lung conditions when inhaled, or systemic infections when ingested by patients with impaired immune systems. Current therapies can be ineffective at treating NTM respiratory infections, even after a long course or with multidrug treatment regimens. NTM, such as *Mycobacterium avium* subspecies *hominissuis* (*M. avium*), is an opportunistic pathogen that shares environments with ubiquitous free-living amoeba and other environmental hosts, possibly their evolutionary hosts. It is highly likely that interactions between *M. avium* and free-living amoeba have provided selective pressure on the bacteria to acquire survival mechanisms, which are also used against predation by macrophages. In macrophages, *M. avium* resides inside phagosomes and has been shown to exit it to infect other cells. *M. avium’s* adaptation to the hostile intra-phagosomal environment is due to many virulence mechanisms. *M. avium* is able to switch the phenotype of the macrophage to be anti-inflammatory (M2). Here, we have focused on and discussed the bacterial defense mechanisms associated with the intra-phagosome phase of infection. *M. avium* possesses a plethora of antioxidant enzymes, including the superoxide dismutases, catalase and alkyl hydroperoxide reductase. When these defenses fail or are overtaken by robust oxidative burst, many other enzymes exist to repair damage incurred on *M. avium* proteins, including thioredoxin/thioredoxin reductase. Finally, *M. avium* has several oxidant sensors that induce transcription of antioxidant enzymes, oxidation repair enzymes and biofilm- promoting genes. These expressions induce physiological changes that allow *M. avium* to survive in the face of leukocyte-generated oxidative stress. We will discuss the strategies used by *M. avium* to infect human macrophages that evolved during its evolution from free-living amoeba. The more insight we gain about *M. avium’s* mode of pathogenicity, the more targets we can have to direct new anti-virulence therapies toward.

## 1. Introduction

Nontuberculous mycobacteria (NTM) are opportunistic human pathogens [1]. NTM are normal inhabitants of the environment, including in natural water sources as biofilm in the drinking water distribution system; and in the human-engineered environment, in both potting and acidic pine forest soil [2]. Additionally, hospitals and health care facility water systems can be reservoirs for NTM [3]. NTM grow in the habitat they share with humans, such as in the plumbing in buildings, and can be classified based on growth rate. Slow growing NTM, which include *Mycobacterium avium* complex (MAC), take 10–14 days for colony formation on Middlebrook 7H10 agar supplemented with 10% OADC at 37 ℃ whereas rapid growers grow in <7 days [4]. Despite the fact that most NTM are environmental, it is important to consider that based on the NTM species, the source of pathogens can be different [4]. For example, *M. avium* is mostly found in water, in contrast to *Mycobacterium intracellulare*, in which the main source is the soil. Recent work suggests that *M. avium* may also be transmitted from individual to individual, since humans are exposed to pulmonary and extrapulmonary diseases due to NTM [5]. The fact that the infection takes an extended amount of time to manifest itself clinically may explain the difficulty to establish this connection.

In the United States, bacteria belonging to the *Mycobacterium avium* complex (MAC) are responsible for more NTM infections than any other species of mycobacteria [6]. MAC mainly consists of *M. avium, M. intracellular* and *Mycobacterium chimaera* [7]. The *M. avium* species is divided into four subspecies: *M. avium* subsp. *Avium*, *M*. *avium* subsp. *Silvaticum*, *M*. *avium* subsp. *Paratuberculosis* and *M*. *avium* subsp. *Hominissuis* (hereafter *M. avium*). *M. avium* is frequently associated with infection in elderly people, as well as in immunocompromised patients such as HIV-1-infected individuals. In contrast, in healthy persons, the innate immune response is thought to control *M. avium* infection [6]. Immunocompetent patients, however, with underlying chronic lung pathology, such as cystic fibrosis, bronchiectasis and emphysema may develop airway infection due to *M. avium* [6]. 

Following ingestion or inhalation, *M. avium* has evolved diverse strategies to ensure growth and survival within the host niche [8]. Here, we discuss different aspects of *M. avium* and the host interaction, including manipulation of host cell signaling pathways [8,9]. NTM are a diverse group of microbes, suggesting a broad acquisition of genes [5,10]. Since NTM are encountered in a variety of environments, which in most cases they share with other bacteria, including co-existing with environmental protozoa, it is plausible to assume that NTM have over time acquired genes and mechanisms to survive inside host cells, with some of them being to infect and persist inside human host macrophages [7].

We summarize and discuss current information about the interactions between the pathogen and mononuclear phagocytes.

## 2. Bacteria Evolution in Protozoa and Other Inhabitants of the Soil

Free-living amoeba have been feeding on bacteria, fungi and other microorganisms for hundreds of millions of years. Microorganisms, such as *M. avium*, evolved mechanisms to survive phagocytosis by amoeba. In fact, the overlapping bacterial infection in amoeba and mammalian phagocytes has fueled interest about evolutionary implications in both host immune responses and bacterial survival mechanisms. Amoeba-resistant bacteria, such as *Legionella spp*., *Chlamydophila pneumoniae*, *Mycobacterium avium*, *Listeria monocytogenes*, *Coxiella burnetii* and *Pseudomonas aeruginosa*, interact with a number of other microorganisms within protozoa [11]. The paucity of significant *M. avium* transmission between humans suggests that the pathogen is not well adapted to the horizontal transmission, similar to *Mycobacterium tuberculosis.* Yet the conjecture stands that interactions between *M. avium* and its evolutionary host, amoeba, provided the selective pressure for bacterial acquisition of virulence determinants, allowing for successful replication within accidentally encountered mammalian macrophages [10,12]. In addition, recent work has begun to question the inability to transmit from host to host, with demonstration in a *C. elegans* model system that it is possible to occur [13]. Investigating such interactions may increase the understanding of *M. avium* adaptation to macrophages and the human host environment.

Several studies have indicated the potential significance of amoeba as hosts for pathogenic environmental mycobacteria. Free-living amoeba are environmentally ubiquitous and are present in fresh and sea water, man-made water networks and soil, where they prey on microorganisms such as mycobacteria [14]. *M. avium* has been also found residing inside the wall of amoeba cysts, which can be a form of transmission to humans when the amoeba are inhaled or ingested [15]. In fact, *M. avium* grown in amoeba prior to infection of macrophages was shown to enhance virulence in vitro and in mice models [10]. Given that both amoeba and macrophages have the capacity to ingest and kill microorganisms, exposure to similar phagocytic environments may benefit *M. avium* for macrophage infection, which is further discussed later in this review. Considering these studies and the environments shared between amoeba, NTM and water resources for humans, it is therefore not very surprising to observe healthcare-acquired infections by amoeba-resistant NTMs, such as *M. avium* [3]. 

Understanding the genealogical history of *M. avium* can elucidate the role that amoeba may have played in shaping mycobacterial virulence factors that are significant for survival in host cells. As it stands, the relationship between virulence characteristics, clinical isolates and genetic phylogeny of *M. avium* are poorly understood. It remains a challenge to succinctly trace the genealogy of *M. avium*, where the composition of chromosomes of clinical isolates is influenced by diverse mycobacterial lineage [16]. MK Shin and SJ Shin provided a comprehensive discussion on the known phylogeny of *M. avium* in their review regarding the role of genetics during *M. avium* complex infection [17]. Advances in molecular analysis and genotyping technology provides the ability to identify known and new species and subspecies within the *M. avium* complex [16]. Comparative whole-genome sequencing of *M. avium’s* complex members revealed a high similarity between subspecies, and analysis of the core genome shared across all *M. avium* complex strains suggested evolution from a common ancestor [18]. Interestingly, sequence diversity varied among the different *M. avium* complex subspecies, where *M. avium* subsp. *hominissuis*, specifically, demonstrated the greatest diversity. It was surmised that the rich sequence diversity is the result of *M. avium*’s intrinsically high rate of horizontal gene transfer over time within myriad environments. Due to *M. avium’s* ubiquitous nature and as prey to free-living amoeba, it would not be surprising for gene acquisition and deletion to occur within amoeba, where the bacteria could selectively alter its gene composition under pressure for survival. Additionally, the presence of exogenous gene fragments or other phagocytized microorganisms in the intracellular environment likely influenced *M. avium* evolution. For example, similar to *M. avium*, *Legionella pneumophila*, the causative agent of legionellosis in humans can co-exist with amoeba [19], showing modes of transmission similar to that of NTMs. Environmental sampling and co-culture studies have consistently demonstrated the mixed presence of *Legionella* and environmental mycobacteria in water systems, both free and as co-infecting protozoa [20,21,22,23]. It is interesting to note that phylogenetic analysis has grouped NTM in a clade with gamma-Proteobacteria *Legionella*, *Tetrahymena thermophila* and *Dictyostelium* *discoideum* [19]. Considering the shared environment among the diverse microorganisms, it is imperative to consider the relationship of the bacteria for possible mechanisms of bacterial pathogenesis. In their cross-genomic, bioinformatic analysis, Lamrabet et al., identified eight mycobacterial genes with close phylogenetic ties outside of Actinobacteria [24]. The authors surmised possible horizontal gene transfers between species, including those of *Legionella* and *M. avium*. Lamrabet et al., suggest a significant connection between pyr-redox and cyst-living mycobacteria, in which pyr-redox may have been acquired within amoeba as a defense against oxidative stress, with a consequent advantage in the interaction against macrophages in the human hosts. Additionally, the authors found that *Legionella* and mycobacteria can live together within amoeba for several days, demonstrating a potential interaction for gene transfer. Other co-cultural studies demonstrated differing growth locations for each pathogen, where *L. pneumophila* only grows intracellularly in the trophozoites of free-living amoeba, while *M. avium* can grow freely on products secreted by amoeba, in addition to intracellular location [23]. 

## 3. Environmental Protozoans Killing of Bacteria

To deal with phagocytized microorganisms, amoeba and macrophages share various killings mechanisms. Resemblance between the phagocytic cells is supported by the high degree of proteome conservation between humans, protozoa and *Dictyostelium* [25]. At least three membrane proteins (Phg1, SadA and SibA) of the actin cytoskeleton are essential in the *Dictyostelium* for cell adhesion and phagocytosis. Phagocytosis is also dependent on integrin. Upon contact, phagosome biogenesis and maturation in amoeba occur similarly to macrophages [26]. As V-ATPases are translocated onto the phagocytic vacuoles, the pathogen is subject to microbicidal acidification of the phagosome mileu. Phagosomes then acquire markers of late endosomes and lysosomes. Additionally, in both amoebas and macrophages, lysosomal hydrolases are delivered to the phagolysosome and are involved in bacterial killing. Of the lysosomal hydrolases, Cathepsin G and elastase have been demonstrated in *S. aureus* and *C. albicans* killing, respectively [25]. The pathogenic *Entamoeba histolytica* in particular is abundant in lysozymes and β-hexosaminidase, which has been implicated in the killing of various intracellular pathogens, including mycobacteria. 

**Figure 1:**
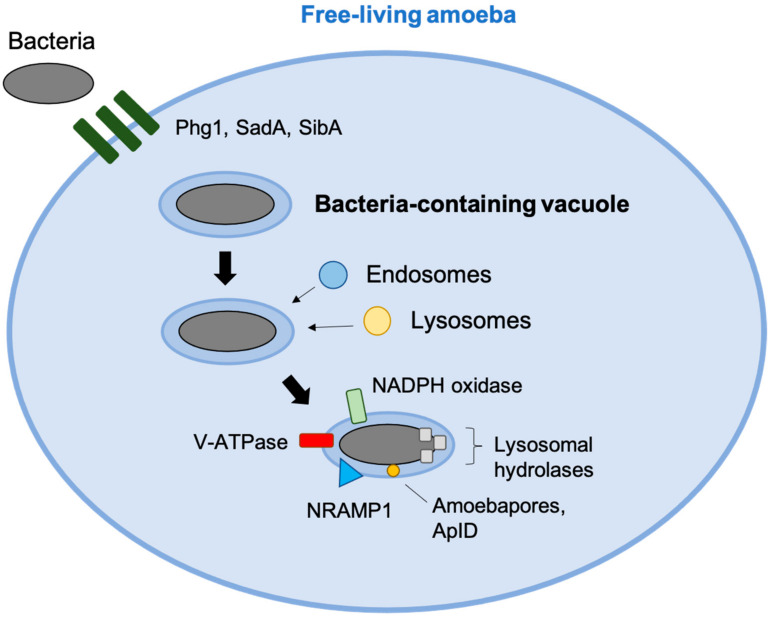
Amoeba defense mechanisms against phagocytized bacteria. Phg1, SadA and SibA are involved in phagocytic uptake. Endosomes and lysosomes fuse with the vacuole as the bacteria-containing vacuole matures over time, contributing to the phagosomes’ arsenal against the bacteria. NADPH generates oxidative burst and works in conjunction with NRAMP1, which depletes Fe(II) and Mg(II) from the phagosomal milieu. V-ATPase acidifies the compartment, and lysosomal hydrolases hydrolyze bacterial membranes. Antimicrobial peptides, such as amaoebapores and AplD are released into the phagosome to permeabilize bacterial membranes.

In *Acanthamoeba castellanii*, there is production of reactive oxygen species (ROS) similar to macrophage oxidative burst [27]. Amoeba-dependent bacterial killing, however, may also be largely independent of ROS. For example, Dictyostelium NADPH-oxidase knockout mutants retain the same ability to kill *Klebsiella pneumoniae* as its wild-type counterpart [28]. This suggests that amoeba may have alternative killing mechanisms. It is known that, for example, phagocytes of individuals with chronic granulomatous disease (CGD), which are unable to produce NADPH-oxidase, are deficient in clearing intracellular bacteria. As of 2006, NADPH-oxidase knockout mutants deficient in *Klebsiella* killing resulted in the identification of two host resistance factor genes: KIL1 and PHG1, which encode a sulfotransferase and a nine transmembrane domain protein, respectively. KIL1 and PHG1 are not necessary to eliminate several other bacteria, which lead to the suggestion that amoebas may have some selection in their killing method against different bacteria (Figure 1).

Protozoa additionally kill phagocytized bacteria by utilizing antimicrobial peptides (AMP). The phylogenetically oldest form of AMP has been found in the pathogenic *Entamoeba histolytica*. *E. histolytica* release lipid-interacting, saposin-like protein (SAPLIP) amoebapore A into phagosomes, which permeabilize the cytoplasmic membranes of bacteria [29,30]. Specifically, three isoforms of *E. histolytica* amoebapore A have been demonstrated to form pores on lipid vesicles and kill Gram-positive bacteria [31]. *D. discoideum* has been recently described to possess 17 genes encoding amoebapore-like peptides (Alp) [32]. Aside from these proteins, AMPs have not been extensively studied on the molecular level in amoebas. It would be an interesting conjecture that intracellular pathogens, such as *M. avium*, have evolved ways to defend themselves against amoeba AMPs that could affect their survival in macrophages. *M. avium’s* competitive survival through its unusual cell wall structure, containing mycolic acids and glycopeptidolipids, may have provided an advantage over other Gram-positive bacteria, preventing pore formation by amoeba AMPs which could be lethal [33].

## 4. *M. avium* and the Phagosome Environment

NRAMP1 is a metal transporter present on phagosomal membranes of macrophages, and homologues have been found on environmental organisms such as *D. discoideum* and *A. castellanii* [25,34]. Its function involves depletion of Fe(II) and Mn(II)-containing phagosomes and confers resistance to bacterial infections including *M. avium* [35]. Loss of NRAMP1 leads to enhanced replication of phagocytized bacteria. NRAMP1 works in conjunction with membrane-bound NADPH oxidase. Hydrogen peroxide reacts with Cu(I) and Fe(II) to generate highly toxic hydroxyl radical and hydroxyl anion. In macrophages only, P-type ATPase transporter ATP7A pumps Cu(I) into the phagosome. Acidification of the phagosome enhances Cu(I) solubility and thereby toxicity. The role of NRAMP1 in macrophages is further discussed in this paper.

There is currently no information on the effectiveness of amoebas or other protozoa AMPs on *M. avium* killing. An *M. avium* transposon library screening revealed several genes resistant to AMP surrogate polymyxin B; mutants for which these resistant genes are knocked out demonstrated *M. avium* attenuation in a macrophages model system and mice [36]. It would be interesting to perform a comparative study on AMP-resistant gene knockout mutants in amoebas, and their effect on intracellular survival. In fact, there is shared SAPLIP homology between *E. histolytica* amoebapore and granulysin of mammalian natural killer (NK) cells, which can independently kill extracellular *M. tuberculosis*, and has activity against *M. avium* in vitro and in vivo [37].

Similar to macrophages and many other organisms, environmental protozoa use autophagy for various homeostatic tasks, such as protein turnover, aggregate degradation, nutrient acquisition during starvation and intracellular pathogen killing (xenophagy) [38]. In their review, Mesquita et al., describe macroautophagy as the best studied, most conserved autophagic process, and the only one described in the model *D. discoideum.* Macroautophagy describes either selective (xenophagy) or non-selective bulk autophagy (starvation) [39]. *D. discoideum* development occurs in the absence of nutrients, rendering autophagy crucial for its survival. Since the amoeba goes through various growth stages and is a ubiquitous organism, it is clear that it must have developed various autophagy mechanisms specific to the developmental stage, availability of nutrients in the environment and presence of intracellular pathogens [40]. Mesquita et al., suggest that the diverse ecologies of protozoa have driven the development of methods to distinguish between food sources and pathogens. On the other hand, microorganisms that are phagocytized by protozoa might have developed ways to survive autophagy that could have possibly contributed to the survival in mammalian phagocytic cells. *L. pneumophila*, for example, has been demonstrated to have autophagy evasion mechanisms that act similarly between amoebas and human macrophages [41]. *M. avium*, which we have discussed as environmental co-inhabitants of *L. pneumophila* within amoeba, also evades autophagy killing in macrophages. In fact, *M. avium* can utilize macrophage autophagy to thrive within the phagocytic cell (under review). *M. tuberculosis* has also been shown to process mechanisms to escape autophagy [42].

*M. avium* encounters a high concentration of antimicrobial peptides as it passes through the mucosal barrier, but it is capable of surviving the mucus layers [36,43,44]. used polymyxin B as a surrogate for host antimicrobial peptides to screen mutants susceptible to host antimicrobial peptides [36]. The result showed that most of the identified genes of *M. avium* mutant library were those related to cell wall synthesis and permeability, and most of the mutants identified were also vulnerable to cathelicidin (LL-37) [36]. Therefore, these results suggested that the envelope of *M. avium* is the primary defense mechanism against host antimicrobial peptides [34]. Furthermore, long-chain acyl-CoA dehydrogenase, MAV_3616, was shown to have a significant role in antimicrobial peptide resistance (Table 1) [43]. Another defense strategy to limit bacterial growth is the phagosome formation and fusing with lysosomes that leads to phagosome maturation, providing an acidic environment (pH 4.5–5.0) that is sufficient to eliminate the internalized bacterium [45,46]. Pathogenic mycobacteria, however, prevent phagolysosome fusion [47,48,49]. For example, *M. avium* prevents phagosome maturation and fusion to lysosomes, which prevents the intracellular vacuole from acidifying and killing the bacteria residing there [50,51]. Acid resistance in bacteria is mediated by several mechanisms, including proton extrusion, amino acid decarboxylation and cell envelope modification. MAV_2941 is a small protein (73 amino acid) that is exported to the cytoplasm of macrophages by oligopeptide permease A (oppA) [8]. MAV_2941 interacts with the vesicle trafficking proteins syntaxin-8 (STX8), adaptor-related protein complex 3 (AP-3) complex subunit beta-1 (AP3B1) and Archain 1 (ARCN1) in mononuclear phagocytic cells (Table 1) [8]. The binding site of MAV_2941 is structurally homologous to the human phosphatidylinositol 3- kinase (PI3K) [8]. Mutated MAV_2941, where the amino acids homologous to the binding region of PI3K were changed, failed to interact with trafficking proteins involved in reducing *M. avium* survival within differentiated ThP-1 human macrophages [8]. MAV_2928 is essential for survival of the bacterium inside of host macrophages (Table 1) [52,53]. Transposon-based disruption of this gene, which is in the ESX-5 loci, prevented the bacterium from arresting the maturation of the phagosome and led to a decrease in virulence. Phagosomes containing the mutant *M. avium* strain quickly acidified, unlike wild-type *M. avium*. Further investigation revealed that markers associated with phagosome maturation, such as EEA-1 and CREB-1, were present on phagosomes containing the mutant strain, but absent on those containing wild-type *M. avium*, which usually acquire Rab5, but absent on those containing wild-type *M. avium*, which usually acquire Rab5, but not EEA-1 [54]. Furthermore, the calmodulin-like protein MAV_1356 interacts with the human THP-1 macrophages proteins, Annexin A1 and Protein S100-A8, to block phagosome-lysosome fusion [55].

Intracellular *M. avium* vacuoles remain at a pH of 6.5–6.9, although the bacteria can tolerate a pH of 6.0 due to its ability to interfere with the vacuolar ATPase from docking to the phagosomal membrane [60]. In order to limit intracellular replication of pathogenic bacteria, macrophages modulate intracellular iron homoeostasis, thus depriving the mycobacterial phagosome of the iron flux needed for bacterial replication [61,62,63,64]. Natural resistance-associated macrophage protein 1 (Nramp1) is a proton/divalent cation antiporter that has a well-established, unique role in innate resistance to intra-phagosomal pathogens in human and mice [64]. Strains of mice that express the Nramp1D169 allele carry macrophages that are more permissive to *M. avium*, rendering the mice to be highly susceptible to *M. avium* infections. On the other hand, Nramp1G169 strains of mice are quite resistant and can control *M. avium* infections [60]. Humans rarely harbor mutations in the coding region of the *Nramp*1 gene, which may explain the infrequency of *M. avium* infection in immunocompetent individuals. 

**Figure 2:**
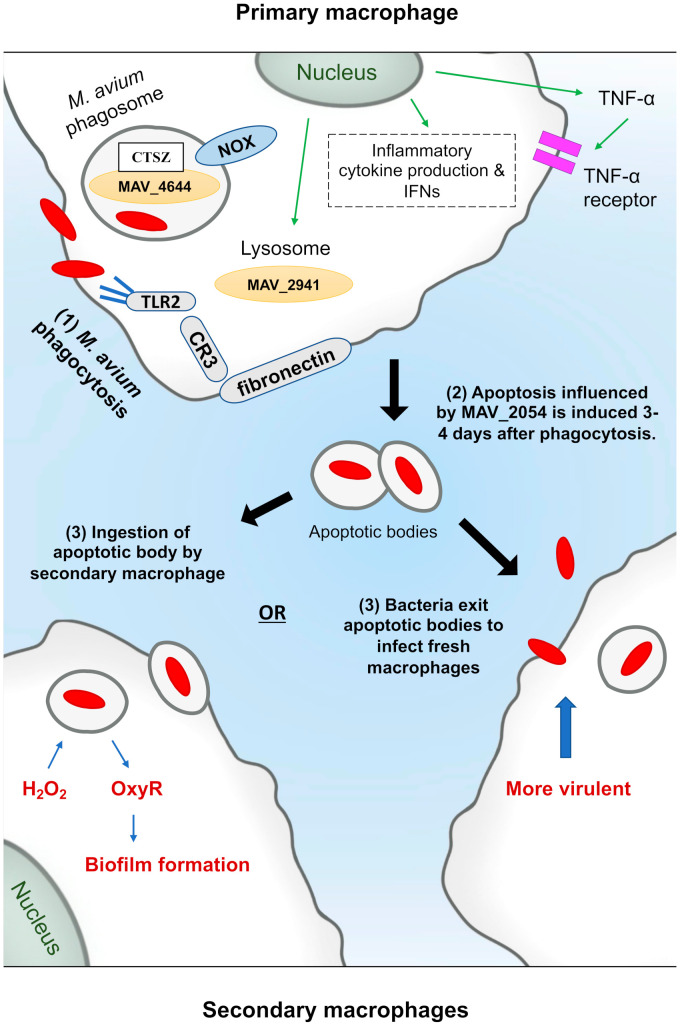
*M. avium* interactions with macrophage and methods for dissemination via apoptosis. *M. avium* is ingested by macrophages via phagocytosis using different receptors as shown, such as CR3. It can signal through toll-like receptor 2 (TLR2), leading to cytokine production and activation of signal transduction pathways, such as the mitogen activated protein kinases (MAPK) and nuclear factor-κB (NF-κB), resulting in further cytokines production. MAV_4644 protein interacts with cathepsin Z (CTSZ) in the phagosome to protect *M. avium* from rapid macrophage killing. MAV_2941 protein is secreted in the cytoplasm to inhibit the fusion of lysosomes with the mycobacterial vacuole. After four days apoptotic bodies are formed, and MAV_2054 contributes to this by targeting mitochondria. Some other bacteria remain inside the apoptotic body, which are taken up by secondary macrophages. Some bacteria then escape both the vacuoles and macrophages to become extra-cellular, and infection can again occur. However, the escaped *M. avium* undergoes a shift in the mycobacterial phenotype towards pathogenesis, as it has been shown that passage of *M. avium* through macrophages improves its ability to infect subsequent macrophages. Inside the secondary macrophages, *M. avium* responds to hydrogen peroxide by producing hydrogen peroxide inducible gene OxyR (MAV_2838) that regulates KatG, GorA, AhpCF, Dps and Fur, that are involved in the preventive pathway to protect the bacterium from oxidative stress. Additionally, MAV_2838 counteracts the effect of H_2_O_2_ leading to activation of regulators that eventually form biofilms.

It appears that as the last resort, to be able to eliminate the pathogen, *M. avium*-infected macrophages undergo apoptosis (a programmed form of cell death). Both murine Raw 264.7 and human THP-1 macrophages have similar amounts of apoptosis triggered by live *M. avium* [51]. In murine macrophages, apoptosis is triggered by the putative cysteine synthase A protein, MAV_2052, which induces apoptosis by TLR-4-dependent ROS production [65]. Whereas the secreted protein, MAVA5_06970, was shown to induce apoptosis in THP-1 macrophages, as well as in vivo [66]. The apoptosis reaches the greatest level five days after infection by tumor necrosis factor TNF α and Fas, leading to the delivery of *M. avium* to the cytoplasm after rupturing the vacuole membrane [49]. *M. avium*-triggered apoptosis is attenuated in the presence of interleukin 10 (IL-10), a TNF-α antagonist [67]. Although apoptosis is a common effect of *M. avium* infection, *M. avium*, however, can survive in apoptotic macrophages and infect new host macrophages. Early et al., proposed a model for the spread mechanism of *M. avium* [49]. Briefly, the model includes: 1. Phagocytosis of *M. avium* by macrophages. 2. Apoptosis induction. 3. *M. avium* can be killed, escape the apoptotic bodies or remain in the apoptotic bodies allowing the process of dissemination upon ingestion by new macrophages. Even apoptotic macrophages failed to control *M. avium* dissemination, which suggests that the immune mechanisms of the host and bacterial strategies for survival are multiplex (Figure 2). 

## 5. *M. avium* Infection of Macrophages: ROS Killing Mechanisms

*M. avium* is a facultative intracellular pathogen that infects and replicates within numerous protozoa species and different mammalian cells, such as macrophages, where it can establish a long-term infection [68]. *M. avium* infects various epithelial cells of the respiratory and gastrointestinal systems as well [69]. Similar to amoeba, macrophages are professional phagocytes and share several intracellular pathogen killing mechanisms. As with amoeba, first contact with the pathogen has a high impact on the eventual outcome of infection. *M. avium* as well as *M. tuberculosis* use complement and other receptors, such as CR3 and CR4 mannose receptors to enter macrophages via phagocytosis [70]. β2-integrin (CD18)-deficient mice, lacking the complement receptors CR3 and CR4, develop *M. avium* infection similarly to the immunocompetent control mice [71], suggesting that the use of these receptors might be redundant or not used by the bacteria in vivo. In fact, studies have also demonstrated that phagocytosis in vivo is independent of complement receptors [71,72]. *M. avium* has acquired genes from proteobacteria most likely co-existing in environmental amoebas, that are associated in vivo with the uptake by macrophages [11]. Interestingly, this *M. avium*-specific pathogenicity island, when mutated, decreases the efficiency of uptake by macrophages in vivo. The genetic island encodes for proteins that allow *M. avium* to enter both environmental amoebas and mammalian macrophages, triggering cytoskeleton rearrangement and phosphorylation of glyceraldehyde 3-phosphate dehydrogenase (GAPDH).

Macrophages’ activation takes place after secretion of pro-inflammatory cytokines, such as IL-12, and IL-23 [73]. In parallel, antigen presenting cell processing leads to interferon-gamma (IFNγ)-producing T helper 1 (Th1) cells, and subsequent activation of macrophages. Additionally, vitamin D3 (VD) also increases the anti-mycobacterial activity of human monocyte-derived macrophages (MDM), and leads to the expression of antimicrobial peptides (AMPs), such as alpha-defensins, beta-defensins and cathelicidin (LL-37). These peptides exhibit broad-spectrum activity against Gram-positive and Gram-negative bacteria, and play important roles in innate immunity [74].

Macrophages employ reactive oxygen species (ROS) as well as reactive nitrogen species (RNS) that are created by the interaction of chemical radicals in the phagosome, in order to kill microbes by targeting protein thiols and metal centers and blocking essential microbial physiological processes, such as respiration and DNA replication [75]. The amount of ROS that is produced is greater in neutrophils than in macrophages, and macrophages generally produce considerably more RNS than neutrophils [76]. However, in vitro studies using chemical inhibitors have shown that the restriction of growth of many isolates of *M. avium* is not dependent on reactive oxygen species or nitric oxide [77]. The intrinsic resistance of *M. avium* to nitric oxide and reactive oxygen species could be due to its thick and waxy cell wall that contains mycolic acids, allowing them to adapt to the nitrite rich environment that is inhabited by *M. avium* when in the soil.

Upon stimulation, macrophage GTPases are activated, which are responsible for the recruitment of NADPH phagocyte oxidase [78]. NADPH oxidase complex has an important role in the early host response and is composed of two membrane proteins, gp91-phox and p22-phox, and three cytosolic proteins, p47-phox, p67-phox and Rac, which assemble after phagocytosis, thereby forming an active enzymatic complex producing superoxide anion and downstream generating hydrogen peroxide along with the formation of hydroxyl radicals [79]. The use of p47phox-deficient animals showed that the oxidative burst is also not required for the control of *M. avium* infection [80].

As an environmental pathogen, *M. avium* may be exposed to photochemically generated superoxide radicals in surface water that is exposed to sunlight [81]. Furthermore, phagocytosis triggers the increase in oxygen consumption by macrophages, resulting in the reduction of molecular oxygen and the massive release in the phagosomal compartment of toxic by-products, such as superoxide radicals, hydroxyle radicals and hydrogen peroxide [82]. Therefore, the genes that undergo transcriptional activation as a result of the oxidative burst encode for proteins that are important for defending *M. avium* from host killing by reactive oxidants. The oxidative stress response to peroxide is primarily mediated by the regulated expression of OxyR [83]. In all the members of *M. tuberculosis* complex and in *M. smegmatis*, oxyR gene is inactivated and represents a pseudogene. In *M. avium* and *M. leprae*, the oxyR gene is active [84]. OxyR is a redox sensing protein belonging to the LysR family of transcription regulators [85]. MAV_2838 is annotated as a homologue to the OxyR transcriptional regulator, based on 38% identity and 53% similarity at the peptide level by BLASTP analysis [86]. The conformation change in the protein resulting from the disulfide bond formation upon oxidation of the two thiol groups by H_2_O_2_ appears to be responsible for the activation of OxyR. Once activated, OxyR positively regulates a group of proteins, such as KatG, GorA, AhpCF, Dps and Fur, which collectively form a part of the preventive pathway to protect the *M. avium* from oxidative stress. KatG is also used by *M. tuberculosis* to protect cells from the damaging effects of H_2_O_2_. Being the only catalase/peroxidase (bifunctional) in *M. tuberculosis*, KatG plays an important role in the physiology and pathogenesis of the bacteria by catabolizing peroxides formed during phagocyte oxidative burst, thus antagonizing the host immune mechanism [87]. *Mtb* lacking *katG*(*Mtb*Δ*katG*) exhibited no catalase activity and was hypersusceptible to H_2_O_2_ in culture [88]. The mutant grew normally in macrophages from NOX2 deficient mice (gp91^phox −/−^). However, *Mtb*Δ*katG* did not grow in wild-type (wt) and iNOS^−/−^ macrophages. In addition to this, *Mtb*Δ*katG* was virulent in mice lacking NOX2, but attenuated in wt and iNOS^−/−^ mice. Viability of *Mtb*Δ*katG* declined rapidly between 2 and 4 weeks, but then remained stable until about 10 weeks post infection when the mutant resumed growth. Collectively, this work demonstrates that *Mtb’s* catalase contributes to virulence in a host that is capable of generating ROI. A previous study has measured the hydrogen peroxide (H_2_O_2_) response in *M. avium* and found that 5 mM and 50 mM of H_2_O_2_ induces biofilm formation in a dose-dependent manner [86]. H_2_O_2_ is sensed by oxyR, which leads to the induction of alkylhydroperoxide reductase and eventually biofilm formation [83]. Alkylhydroperoxide reductase (*ahpC*, MAV_2839) is a protein that catalyzes peroxide reduction, and is a known surface protein of *M. paratuberculosis*, *M. smegmatis* and *B. subtilus* (Table 1) [89]. It is also known to play a role in isoniazid resistance, which is an antibiotic commonly used in the treatment of tuberculosis [57]. Another surface protein is the isocitrate lyase, MAV_4682, which under nutrient limited conditions utilizes fatty acid and acetate as a basic carbon source (Table 1) [57]. In addition to the OxyR regulon, *M. avium* has developed specific enzymatic pathways to maintain the balance of H_2_O_2_ inside the cell. For example, superoxide dismutase (*sodA*, MAV_0182) is a surface exposed protein that catalyzes the dismutation of the superoxide radical to hydrogen peroxide and molecular oxygen (H_2_O_2_ and O_2_). SODs are important for virulence in several bacterial pathogens, including *Helicobacter pylori* [90], *Salmonella typhimurium* [91] and *Yersinia enterocolitica* [92]. SOD is not membrane permeable, and thus superoxide dismutase can only confer resistance to proximally generated superoxide [93]. *SodA*, MAV_0182 activity is increased upon phagocytosis by macrophages, resulting in bacterial survival despite increased inflammatory cytokine production and increased H_2_O_2_ production by the macrophages [57]. *M. tuberculosis* contains two genes encoding superoxide dismutases, *sodA* and *sodC*. SodA, which uses iron, may compensate for SodC to protect against the respiratory burst in naïve macrophages and during mouse infections [94]. SodC is a Cu,Zn superoxide dismutase localized to the mycobacterial cell envelope [95]. A lack of sodC increases susceptibility of Mtb to superoxide alone, as well as to the combination of superoxide and NO and to killing by IFNγ-activated murine peritoneal macrophages [96]. *MtbΔsodC* was able to survive in resting wt macrophages and IFNγ-activated NOX2-deficient macrophages, demonstrating that the Cu,Zn superoxide dismutase contributes to Mtb’s resistance against oxidative burst products generated by activated macrophages. SodC transposon mutants were, however, not attenuated in mice up to 60 days post infection [96,97]. The importance of the Cu,Zn superoxide dismutase for mycobacterial survival during infections has been assessed in macrophages and neutrophils. Monocyte-derived macrophages were infected with the wild-type and MAV_2043 (Cu-Zn-SOD), and a number of intracellular viable bacteria were determined after 1 and 2 h. The deficiency in MAV_2043 (Cu-Zn SOD) had a small effect on the survival of *M. avium* in macrophages. However, when neutrophils were infected (to mimic the initial phase of the infection in vivo), the absence of superoxide dismutase on the surface of *M. avium* was associated with a significant decrease in bacterial viability (Table 1) [58]. Li et al., (2010) identified genes that inhibit phagosome lysosome fusion, thus were related to resistance to oxidative stress in macrophages, and all of the KO strains for these genes were attenuated in the early stages of infection in mice [51]. MAV_4264, which has high homology with the bacterial regulatory protein TetR domain, was shown to regulate the inhibition of acidification by other genes (MAV_2450, MAV_4292 and MAV_4012) (Table 1) [53]. Thus, it was observed that several hypothetical proteins, in addition to SOD, were related to resistance to superoxide anion and reactive nitrogen intermediates [53]. Even with all of the diverse defenses *M.* *avium* possesses to protect its macromolecules from oxidation, it is inevitable that proteins will be oxidized. *M. avium* contains several protein repair mechanisms that allow for the regeneration of proteins instead of having to degrade and replace proteins, a time- and energy-consuming process. To repair the oxidized protein, *M. avium* utilizes enzyme systems consisting of thioredoxin (TrxA) and thioredoxin reductase (TrxB) [98].

## 6. *M. avium* and Macrophages: RNI Killing Mechanism

*M. avium* displays increased virulence during infections in wild-type mice when compared to nitric oxide synthase (NOS2) knockout mice [99], despite of the subsequent action of inducible nitric oxide synthase (NOS2) to control bacterial replication [100]. Additionally, an initial report by Doherty and Sher showed that NOS2-deficient mice were not affected in their susceptibility to *M. avium* [101]. In vitro studies using macrophages from NOS2-deficient mice showed that these macrophages were as successful in controlling *M. avium* upon activation as controlling macrophages from wild-type mice [77]. A subsequent study confirmed that such was the case during the early time points of infection, and that at later stages NOS2-deficient mice were even more resistant to infection [102]. The mycobacterial response to NO within the host macrophage plays an important role in the survival and replication of the pathogen. Notably, *M. avium* displays increased virulence during infections of wild-type mice than when infecting NOS2 knockout mice [77,103]. In this study, the observation was validated using an in vitro model that showed that murine macrophages stimulated with IFN-γ and producing NO are permissive to intracellular growth of *M. avium*, and that intracellular growth was abrogated if the macrophages were treated with an inhibitor of NO production or if IFN-γ was suppressed.

Within the host phagosome, mycobacteria are exposed to the cytotoxic effects of NO produced by the host. NO can be converted to mycobactericidal RNS, such as nitrate or nitrite. Hence, NO has a significant role in the protection of diseases caused by mycobacteria in healthy individuals [104]. In immunocompromised individuals, however, the killing effects of NO are diminished. Upon exposure to NO, *M. tuberculosis* can go into dormancy, whereas *M. avium* survives exposure to RNS without effect on its activity and viability [77]. Denitrification is the reduction of nitrate or nitrite that can be utilized by bacteria when oxygen is not readily available. In anerobic conditions or environments in which nitrogen substrates are abundant, mycobacteria may use denitrification to continue respiration and survive within phagosomes, which could explain *M. avium’s* ability to survive NO-mediated killing.

Exposure to NO inhibits *M. tuberculosis* respiration and reversibly slows growth and replication. However, viability is not significantly affected even at high concentrations [105]. Depletion of oxygen and non-toxic doses of NO was found to induce expression of 48 genes within the dormancy survival regulator DosR in Mtb, that promotes dormancy within macrophages, including nitrate transporter narK2 and respiratory nitrate reductase narGHJI. Dormancy increased survival of in vitro *M. tuberculosis* in latent models, with decreased sensitivity to antibiotics. Similarly, in vitro *M. avium* entered a non-replicated state in the face of acidic, hypoxic and nutrient-scarce conditions with decreased sensitivity to antibiotics [106]. 

Bacteria use alternative terminal electron acceptors in the absence of oxygen. Nitrate is second best to oxygen as a terminal electron acceptor. Addition of exogenous nitrate protected *M. tuberculosis* from hypoxic acidic killing [107]. Nitrate reductase is membrane-bound on *M. tuberculosis*, and confers acid resistance in the face of hypoxic conditions. *M. tuberculosis* exports nitrate product nitrite into extracellular milieu. Regardless of the host producing iNOS or not, or the bacteria being in hypoxic conditions, *M. tuberculosis* is found to use nitrate for respiration and produce nitrite in large amounts. This builds up ATP reserves that can be used upon reactivation of *M. tuberculosis* when infecting other hosts. In response to the produced endogenous nitrite, *M. tuberculosis* may assume a dormant state [108]. *M. avium* can use nitrate and nitrite as nitrogen sources, but does not grow well with nitrite [109]. However, there is evidence of a rapid reduction of nitrite but slow reduction of nitrate. The presence of nitrite reductase may contribute to *M. avium* survival and resistance to dormancy, unlike *M. tuberculosis*, which becomes latent when exposed to nitrite. This could explain *M. avium*’s survival in environments with increased levels of nitrate and nitrite, as seen in CF patients infected with the pathogen.

On the other hand, in the presence of oxygen, the dimeric hemoglobin HbN of *M. tuberculosis* putatively acts as a nitric oxide dehydrogenase that metabolizes NO to nitrate [110]. *M. tuberculosis* HbN expression in *E. coli* and *M. smegmatis* protected them from NO-induced growth inhibition, with a 100-fold increase in NO metabolism exhibited in the latter. This demonstrates a possible mechanism for NO-mediated killing evasion for *M. tuberculosis*. Genome sequences revealing orthologues of HbN have been found in several other mycobacterial species, including a 79% orthologue found in *M. avium* [111]. This suggests an important function of HbN in mycobacterial metabolism, and may assist in the defense against host NO-mediated killing for *M. avium* as well. To our knowledge, there are currently no studies investigating a possible role of HbN in *M. avium*. It would be interesting to determine the role of HbN as an alternative detoxification factor, where *M. avium* can subsequently utilize nitrate by-products for cellular respiration and persistence within macrophages. 

The murine macrophages untreated with IFN-γ and infected with *M. avium* were capable of robust production of NO, near to the levels of IFN-γ-activated cells. Despite this, these cells were not permissive to *M. avium* growth, indicating a potential role of NO as a signal molecule to the pathogen. Regardless, *M. avium* survives and replicates under nitrosative stress. Lewis et al., identified *MAV_*4644 by screening an *M. avium* transposon library for mutants that are susceptible to killing by reactive nitrogen intermediaries (Table 1) [9]. *MAV*_4644: Tn gene knockout clone was also significantly attenuated in growth within both murine macrophages and ThP-1 human macrophages, suggesting its role in human pathogenesis. Complementation of the mutant restored the wild-type phenotype. The *MAV*_4644 gene is a putative ADP-ribosyltransferase (ADPRT), which is partially well conserved among pathogenic mycobacteria. MAV_4644_CTD interacts with host protein cathepsin Z in an immunoprecipitation assay. MAV_4643 and MAV_4642 recombinant proteins also interacted with cathepsin Z. This could indicate that the proteins of the operon work together to interact with the host protein. Cathepsins are lysosomal peptidases and operate in several cell functions such as protein processing, pathogen killing, antigen presentation and apoptosis within macrophages [112]. Cathepsin Z has been positively identified as a protein of the early endosome, and such proteins are accessible to the mycobacterial vacuole [113]. The knock-down of cathepsin Z in ThP-1 human macrophages rescued the attenuated phenotype of MAV_4644: Tn to near wild-type levels of survival [9]. The data suggest cathepsin Z is involved in early mycobacterial killing within host macrophages, and virulence factor MAV_4644 protein protects the pathogen from this process [11]. Although the purified cathepsin Z by itself does not have any killing effect on *M. avium*, it contributes to bacterial killing in the presence of NO [9,112].

### M. avium and Methylation of DNA in Macrophages

**Figure 3:**
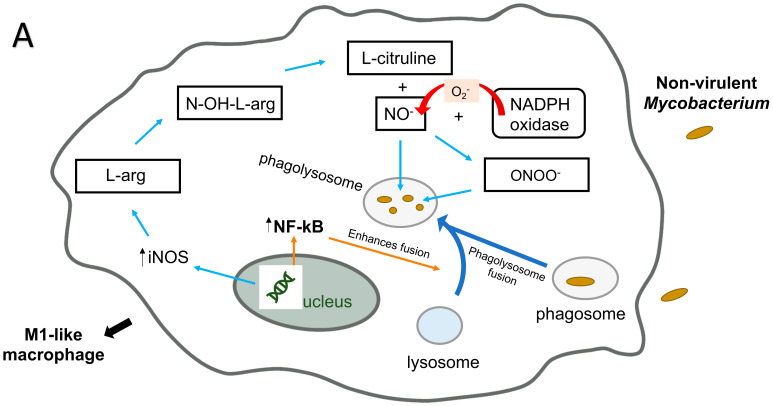

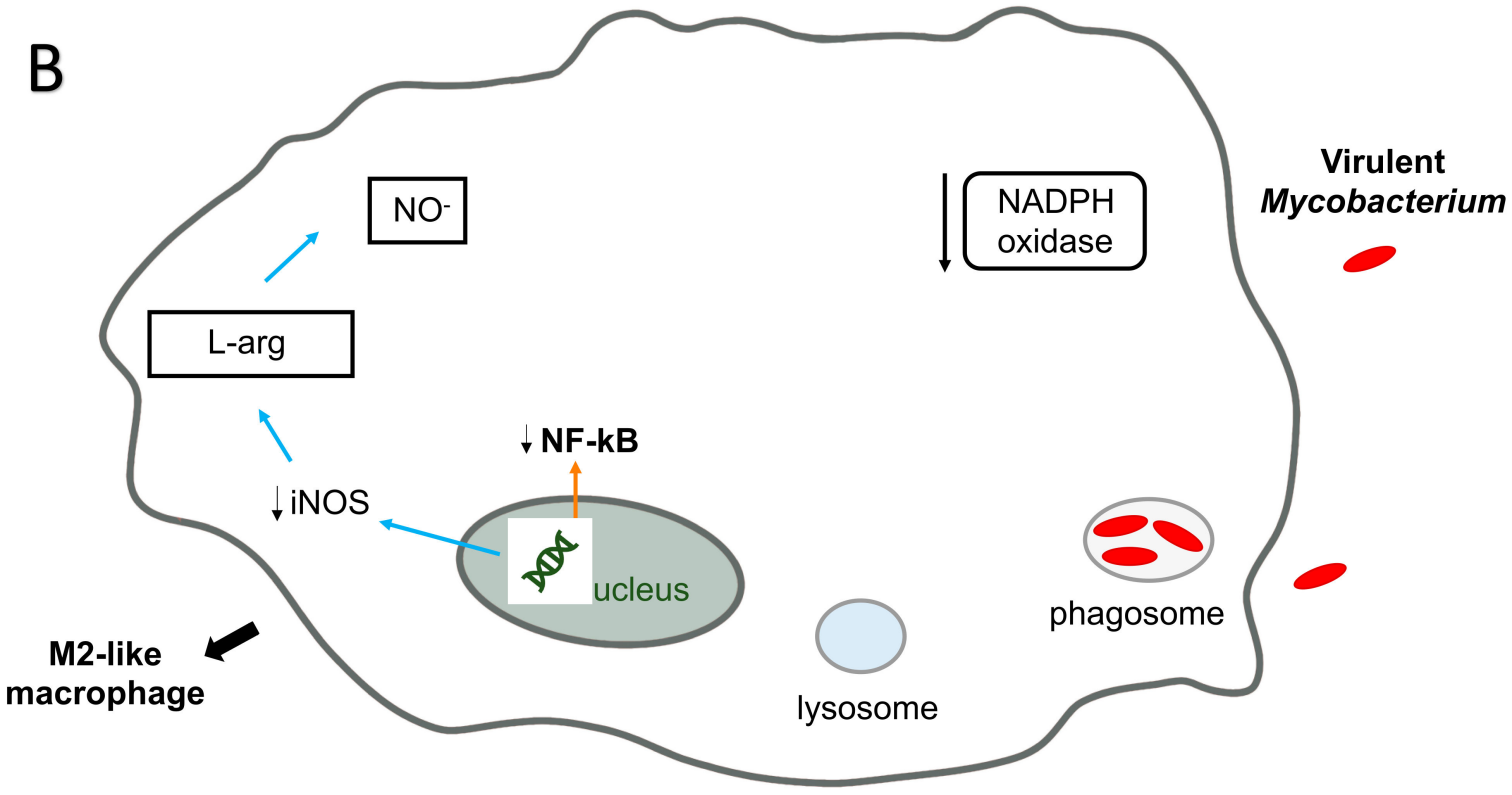
Interaction of *M. avium* and non-virulent mycobacteria with macrophage killing mechanisms. Infection of macrophages with the non-virulent mycobacteria induces the M1-like macrophage phenotype (**A**). The phagocyte has high antimicrobial capacity and is proinflammatory, producing cytokines, such as TNFα, IL-1β, IL-6, IL-12 and IL-23 and is more efficient in the production of antimicrobial molecules, such as nitric oxide and reactive oxygen intermediates. The vacuoles containing non-virulent mycobacteria fuse with lysosomes to form the phagolysosome (Figure 3A). Non-virulent mycobacteria are readily killed in the phagolysosomes, which are rich in hydrolytic enzymes, and have extremely low pH and possess several bactericidal peptides. Infection of macrophages with the virulent mycobacteria (*M. avium*) induces the M2-like macrophage phenotype (**B**). The phagocyte has low antimicrobial capacity and is anti-inflammatory, producing cytokines such as IL10. Vacuoles containing pathogenic mycobacteria (*M. avium*) do not fuse with lysosomes (Figure 3B). *M. avium* prevents phagosome maturation and fusion with lysosomes, which prevents the intracellular vacuole from acidifying and killing *M. avium* in the phagosome.

Recently, it was discovered that virulent isolates of *M. avium*, in contrast to attenuated ones, and *Mycobacterium smegmatis*, induce phosphorylation of a methionine adenosyltranferase II beta, an enzyme that catalyzes the biosynthesis of S-adenosylmethionine, a DNA methyl donor, right after uptake of the bacterium by macrophages. Once activated, methyltranferases methylate the DNA, preventing transcription. The particular one phosphorylated by virulent *M. avium* has been shown to block the synthesis of inflammatory cytokines [114]. In fact, macrophage infected with virulent *M. avium* strain, but not with the attenuated strain, differentiate in M2 macrophages, while infection with attenuated strains are associated with the M1 macrophage phenotype. Other pathogens utilize a similar strategy for survival inside phagocytic cells, with DNA methylation and shift of macrophage phenotype to M2 macrophage, a non-inflammatory phenotype [115,116] (Figure 3A,B).

## 7. Using the Current Understanding to Benefit Treatment

The underlying mechanisms for *M. avium* pathogenesis from environmental sources of infection to their survival strategies within host cells have complicated *M. avium* therapy. As a result, *M. avium* requires prolonged treatment for 15 to 18 months, which poses challenges for patient adherence, thereby contributing to the emergence of antibiotic resistance. In addition, because *M. avium* changes their metabolic and replication dynamics in the host environment, it becomes more persistence and drug tolerant [117]. To identify suitable candidate targets, our laboratory has started to use flux balance analysis (FBA). The use of FBA is well-established and allows insights into the metabolic pathways chosen by the organisms under different environmental conditions, such as in low oxygen, low nutrient conditions. This is because under these unfavorable environmental conditions, *M. avium* maintains a non-replicate state of infection by expressing and synthesizing specific sets of proteins. Identifying these proteins that are expressed within different environmental stresses will help to understand what metabolic pathway *M. avium* uses in different host environments, and this will bring enormous potential to medicine and patient’s health.

## 8. Conclusions

**Figure 4:**
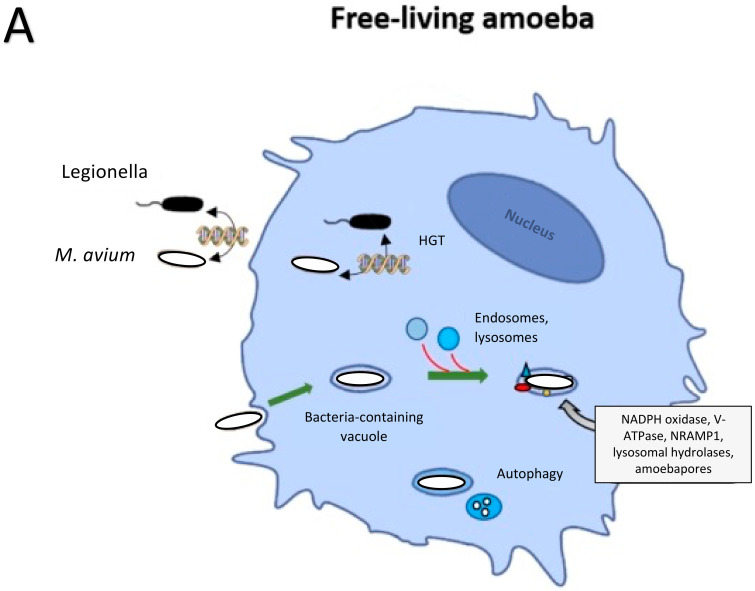

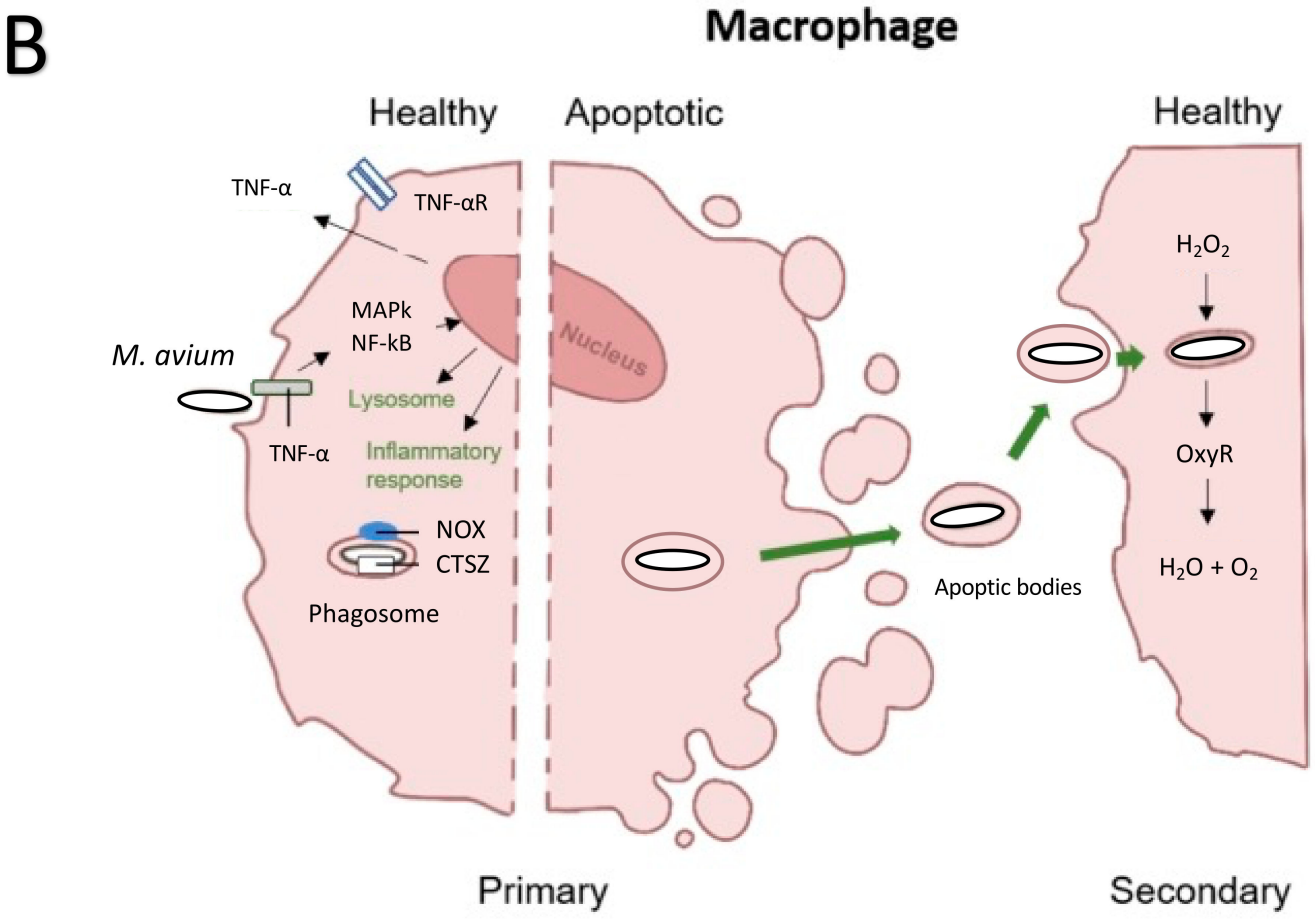
Summary of bacterial killing mechanisms and host-pathogen interactions for phagocytic cells, amoeba and macrophages (**A**) amoeba and macrophages share various killing mechanisms against pathogens. As the model organism for studying phagocytic uptake in macrophages, it is known that phagosome maturation after uptake of environmental microorganisms occurs similarly between amoeba and macrophages. Due to the ubiquitous nature of *M. avium* and that amoeba is its evolutionary host, it is highly likely that *M. avium* obtained survival mechanisms within amoeba that contributes to survival within macrophages. (**B**) Macrophage phagosomes containing *M. avium* are not able to fully mature, allowing the bacteria to persist before apoptotic spread to other macrophages.

*M. avium* is a robust environmental pathogen that resides primarily in environmental sources, therefore, the cellular processes that it utilizes to infect amoeba and macrophages are similar (Figure 4). These bacteria are mostly responsible for lung infection, as well as disseminated disease, as seen in immunocompromised individuals. *M. avium* is resistant to many antibiotics and toxic molecules due to the impermeability of its thick cell wall that contains diacylated and triacylated lipoproteins. The protection of its intracellular niche inside host macrophages makes the infections difficult to treat. Overcoming the bactericidal properties of macrophages is key to avoid certain host defenses that aid in intracellular killing. Among the survival strategies discussed here are arresting the maturation of the phagolysosome as well as utilization of nitric oxide for survival and replication. Several studies have confirmed the role of nitric oxide for *M. avium* virulence. Different from *M. tuberculosis*, the inactivation of induced NO has no effect on *M. avium*, as it replicates in nitric oxide producing cells and is less virulent when inducible nitric oxide synthesis is suppressed in mice. Because the initial stages of the infections seem to be key in *M. avium* pathogenesis by modulating the macrophage to allow for more robust intracellular growth and inducing anti-inflammatory cytokines that could potentially interfere with inflammatory processes, we have discussed the antimicrobial peptides to increase our understanding of how *M. avium* interacts and infects epithelial cells and also eventually macrophages [118]. Nonetheless, while little is known about the interdependence of stress resistance observed for RNI and ROI defense, their requirement for *M. avium* virulence has been demonstrated.

## Figures and Tables

**Table 1:** Summary of the virulence-related genes of *M. avium* discussed in this review.

Gene Name	Description	Function in *M. avium*	Virulent Mechanisms	Reference
MAV_3616	Long-chain acyl-CoA dehydrogenase	Catabolism of fatty acid and amino acids	Antimicrobial peptides resistance.	[36]
MAV_2941	Hypothetical protein	Hijacking host binding protein	Inhibition of phagosome-lysosome maturation by mimicking the binding site of host vesicle trafficking proteins.	[8]
MAV_2928	PPE25_MAV	Secreted protein, type VII secretion system	Inhibition of phagosome-lysosome maturation.	[52,56]
MAV_2839	Alkylhydroperoxide reductase	Catalyzes peroxide reduction	Resistance to oxidative stress, such as reactive oxygen intermediates.	[57]
MAV_4682	isocitrate lyase	Metabolism, glyoxylate shut	Resistance to oxidative stress.	[57]
MAV_2043	Cu-Zn-SOD	Catalysis, superoxide dismutase	Resistance to oxidative stress phagosome acidification.	[58]
MAV_4264	Hypothetical protein	Unknown function, homology with bacterial regulatory protein TetR	Resistance to both oxidative stress and phagosome acidification.	[59]
MAV_4644	ADP-ribosyltransferase	Interfering with host cathepsin Z protein	Resistance to oxidative stress, such as nitric oxide. Resistance to phagosome acidification	[9]

## Data Availability

Not applicable.
